# Urinary C-Peptide Measurement as a Marker of Nutritional Status in Macaques

**DOI:** 10.1371/journal.pone.0018042

**Published:** 2011-03-30

**Authors:** Cédric Girard-Buttoz, James P. Higham, Michael Heistermann, Stefan Wedegärtner, Dario Maestripieri, Antje Engelhardt

**Affiliations:** 1 Jr. Research Group Primate Sexual Selection, Reproductive Biology Unit, German Primate Centre, Goettingen, Germany; 2 Courant Research Centre Evolution of Social Behaviour, Georg-August University, Goettingen, Germany; 3 Institute for Mind and Biology, University of Chicago, Chicago, Illinois, United States of America; 4 Reproductive Biology Unit, German Primate Centre, Goettingen, Germany; Boston University, United States of America

## Abstract

Studies of the nutritional status of wild animals are important in a wide range of research areas such as ecology, behavioural ecology and reproductive biology. However, they have so far been strongly limited by the indirect nature of the available non-invasive tools for the measurement of individual energetic status. The measurement of urinary C-peptide (UCP), which in humans and great apes shows a close link to individual nutritional status, may be a more direct, non-invasive tool for such studies in other primates as well and possibly even in non-primate mammals. Here, we test the suitability of UCPs as markers of nutritional status in non-hominid primates, investigating relationships between UCPs and body-mass-index (BMI), skinfold fatness, and plasma C-peptide levels in captive and free-ranging macaques. We also conducted a food reduction experiment, with daily monitoring of body weight and UCP levels. UCP levels showed significant positive correlations with BMI and skinfold fatness in both captive and free-ranging animals and with plasma C-peptide levels in captive ones. In the feeding experiment, UCP levels were positively correlated with changes in body mass and were significantly lower during food reduction than during re-feeding and the pre-experimental control condition. We conclude that UCPs may be used as reliable biomarkers of body condition and nutritional status in studies of free-ranging catarrhines. Our results open exciting opportunities for energetic studies on free-ranging primates and possibly also other mammals.

## Introduction

Nutritional status significantly influences an individual's daily activities, ranging from the maintenance of basal body functions to the behavioural strategies that ensure survival and successful reproduction. In the long-term, it influences all aspects of animal life-history from growth, life-time reproductive output to longevity [1]. Studies on individual energetic status therefore form an important part of biological research areas such as ecology, behavioural ecology and reproductive biology (e.g. [2]–[6]). Direct measurement of energetic status has, however, been limited so far for wild and/or free-ranging animals by the lack of suitable non-invasive tools.

In non-human primates, for example, most studies have so far used very indirect or invasive methods for the measure of individual nutritional status such as: 1) the visual estimation of body fat and/or condition (e.g. [7]–[8]), which is subjective, susceptible to inter-observer inconsistencies, and only possible when differences in nutritional status are extreme enough to recognise them visually; 2) the weighing of animals, which is usually difficult in the wild, either requires trapping or darting of animals (inducing stress and potentially altering natural behaviour and metabolism [9]–[14]) or includes baiting scales with food [15]–[16], which interfers with studies of nutritional status. These problems are particularly problemmatic when measurements need to be systematically repeated, and for arboreal animals; 3) behavioural observation, in which calorific intake is estimated (e.g. [17]–[18]) or feeding and travelling activity budgets used (e.g. [19]), methods that are labor intensive, imprecise, and have produced results that have proven difficult to interpret [18], [20]–[21]; 4) the assessment of urinary ketones, a measure of fat metabolism with a semiquantitative strip test (e.g. [22]) which allows only very rough quantification of nutritional status and is often not sensitive enough to detect intra- or inter-individual variation [23]–[25]; and 5) the measure of nutritional status through the use of X-rays [26] or doubly-labelled water [27], which cannot be applied to free-ranging or wild animals without capture (see above).

The analysis of urinary C-peptide, which has recently been shown to be closely linked to individual nutritional status in humans and great apes, may offer a method to allow energetic status to be measured more directly and with a higher degree of sensitivity in free-ranging/wild mammals. C-peptide is produced in an equimolar ratio to insulin when the body converts proinsulin to insulin during insulin biosynthesis [28]. In humans, C-peptide is excreted in urine (urinary C-peptide; UCP), with excretion levels correlated with internal C-peptide secretion and insulin production [29]–[30] under normal circumstances, making UCP a useful marker of insulin production in many clinical studies (e.g. [31]–[32]). Increased calorific intake over 24 h periods leads to increases in 24 h UCP excretion [33], and levels of 24 h UCP excretion are positively correlated with body weight [33] and BMI [29]. Recent studies of great apes have demonstrated similar relationships, with UCP levels shown to correlate with serum C-peptide levels and ripe fruit consumption in chimpanzees (*Pan troglodytes*; [34]–[35]), energetic intake estimates and fruit availability in orangutans (*Pongo pygmaeus*; [36]), and body mass in bonobos (*Pan paniscus*; [37]) (see Table 1). In addition, feeding experiments have shown that UCP levels respond to dietary changes, with low levels excreted during fasting, and higher levels during re-feeding [37].

**Table 1:** Summary of the known relationship between Urinary c-peptide levels and different measures of nutritional status for different primate species under different settings.

Species	Setting	Parameters							
		Serum insulin levels	Serum/plasma c-peptide levels	Body			Food Intake	Natural food availability	Ketone bodies
				Mass	Fatness	Mass Index			
Human	NA	(+) [29], [30]	(+) [29.30]	(+) [33]		(+) [29]	(+) [33]		
Bonobo	captive			(+) [37]			(+) [37]		
Orangutan	wild						(+) [35]	(+) [35], [36]	(+) [35]
Chimpanzee	captive		(+) [35]						
Chimpanzee	wild							(+) [34], [35]	
Rhesus macaque	captive	(+) [38]	(+) §	(+) §	(+) [38]		(+)[38] §		
Rhesus macaque	free-ranging				(+) §	(+) §			
Long-tailed macaque	captive		(+) §	(+) §	(+) §	(+) §	(+) §		

(+): positive relationship between urinary c-peptide levels and the parameter under study.

§present results.

NA: Non applicable.

UCPs have also been measured in non-great ape primate species. A previous study investigating behavioural and physiological correlates of obesity in captive rhesus macaques (*Macaca mulatta*) using 12 hour urine collections for UCP measurement found that obese animals had significantly higher UCPs than non-obese ones, and that urinary and serum C-peptide levels were correlated [38]. In a more recent study on colobus monkeys (*Colobus guereza*), scarcity of top folivorous was associated with a decline in UCPs in two lactating females [39]. Collectively, these studies give reason to believe that UCPs are a valuable biomarker of energetic status also in non-hominid primates, if not even in mammals in general. However, a thorough validation of urinary C-peptides as a measure of energetic condition based on analysis of single urine voidings, usually the only type of sample available in the wild, has not yet been performed for non-hominid primate species.

To undertake such a validation, we conducted a study on macaques in order to investigate systematically the general value of UCPs as biomarkers of nutritional status using a dual approach. First we investigated inter-individual relationships between UCP levels and body mass index (BMI), body fatness, and levels of plasma C-peptide in captive rhesus and long-tailed macaques housed at the German Primate Centre (DPZ). In order to test the robustness of the data obtained for these captive animals and thus see whether C-peptide measurements would provide similar results also under field conditions, we repeated this investigation for all the same variables using free-ranging rhesus macaques on Cayo Santiago (CS), Puerto Rico. Second, we carried out a 4-week feeding experiment on DPZ animals to explore the interrelationships between changes in food supply (and thus energy intake), body mass and UCP levels in a more fine tuned way. We predicted that: 1) UCPs would correlate positively with plasma C-peptides, BMI and body fat in both captive and free-ranging animals; 2) UCPs would covary with body mass in response to dietary changes, decreasing during food-reduction, and increasing during re-feeding (as in Bonobos, [37]). These analyses represent the first comprehensive test of the likely utility of urinary C-peptide for assessing energy intake and body fatness in studies of non-hominid primates.

## Methods

### Ethics Statement

The protocol for this study was approved by the government of Lower Saxony, Germany for DPZ animals (permit number: 33.14-42502-04-106/09) and by the Institutional Animal Care and Use Committee, University of Puerto Rico, for CS animals (protocol number: A0100108). All research undertaken adhered to all animal care, legal and ethical requirements of Germany, the United States and Puerto Rico, as well as the Animal Behavior Society (ABS) and Association for the Study of Animal Behaviour (ASAB) "Guidelines for the Use of Animals in Research", and the American Society of Primatologists (ASP) "Principles for the Ethical Treatment of Non-Human Primates". CS animals are free-ranging, while all DPZ animals are housed either as pairs or groups, allowing for social interactions. The vast majority of samples were collected non-invasively (urine samples). Blood samples were collected only once from each individual during the study under sedation by trained veterinarians.

### Study Sites and subjects

The study was conducted between 29^th^ September 2009 and 17^th^ February 2010 on 11 adult (≥5 years of age) captive macaques: 6 males and one female long-tailed (*Macaca fascicularis*) and two males and two female rhesus (*M. mulatta*) macaques housed at the German Primate Centre (DPZ) and, in addition, between 3^rd^ November 2008 and 24^th^ February 2009 on 13 adult free-ranging male rhesus macaques living on the island of Cayo Santiago (CS), 1 km off the coast of Puerto Rico [40]. All captive animals were housed either as same-sex pairs or small same-sex groups in indoor cages, or as single-male-multi-female groups in outdoor enclosures with access to an indoor cage (see Table 2). DPZ macaques are fed twice a day (early morning and noon) with commercial monkey chow supplemented with fruits. The CS macaques are free-ranging and feed on natural vegetation on the island, but are also provisioned daily in the early morning with commercial monkey chow, made available in several feeding corrals. Though food composition varies between individuals and seasons, on average, Cayo rhesus spend 50.2% of their feeding time ingesting monkey chow, and 49.8% on ingesting a variety of natural vegetation [41].

**Table 2:** Species, sex, age, body weight, and housing condition of animals.

Animal	Species	Sex	Age (years)	Body weight (kg)	Housing condition		Used in Experiment
					Group	Space	
2136	*M. mulatta*	Female	8	8.3	Unisex pair	DPZ - Indoor	Yes
2146	*M. mulatta*	Female	8	6.75	Unisex pair	DPZ - Indoor	Yes
14227	*M. mulatta*	Male	9	17.1	Unisex pair	DPZ - Indoor	Yes
12225	*M. mulatta*	Male	9	12.9	Unisex pair	DPZ - Indoor	Yes
12466	*M. fascicularis*	Male	5	4.4	Unisex group of 4	DPZ - Indoor	Yes
12331	*M. fascicularis*	Male	5	4.95	Unisex group of 4	DPZ - Indoor	Yes
12401	*M. fascicularis*	Male	5	5.6	Unisex group of 4	DPZ - Indoor	No
12400	*M. fascicularis*	Male	5	5.1	Unisex group of 4	DPZ - Indoor	No
10778	*M. fascicularis*	Male	9	5.25	Singly caged	DPZ - Indoor	No
10786	*M. fascicularis*	Male	12	12	Single male multi-female group	DPZ - Outside	No
10587	*M. fascicularis*	Female	9	5.1	Single male multi-female group	DPZ - Outside	No
39L	*M. mulatta*	Male	9	10.0	Multi-male multi-female group	CS – Free-ranging	No
83L	*M. mulatta*	Male	9	12.9	Multi-male multi-female group	CS – Free-ranging	No
17K	*M. mulatta*	Male	10	10.2	Multi-male multi-female group	CS – Free-ranging	No
44H	*M. mulatta*	Male	11	13.3	Multi-male multi-female group	CS – Free-ranging	No
61G	*M. mulatta*	Male	12	8.9	Multi-male multi-female group	CS – Free-ranging	No
42F	*M. mulatta*	Male	13	13.5	Multi-male multi-female group	CS – Free-ranging	No
57D	*M. mulatta*	Male	14	10.5	Multi-male multi-female group	CS – Free-ranging	No
03D	*M. mulatta*	Male	14	13.1	Multi-male multi-female group	CS – Free-ranging	No
50B	*M. mulatta*	Male	16	8.8	Multi-male multi-female group	CS – Free-ranging	No
14A	*M. mulatta*	Male	17	9.8	Multi-male multi-female group	CS – Free-ranging	No
T82	*M. mulatta*	Male	19	8.8	Multi-male multi-female group	CS – Free-ranging	No
O15	*M. mulatta*	Male	21	8.3	Multi-male multi-female group	CS – Free-ranging	No
K85	*M. mulatta*	Male	22	13.5	Multi-male multi-female group	CS – Free-ranging	No

### Collection of morphometric data and plasma sample collection

Collection of morphometric data and blood samples of DPZ animals took place during the annual health check of the macaque colony during which animals are anesthetized in the early morning with an intra-muscular injection of ketamine hydrochloride (10 mg/kg) applied by darting the animal. CS animals were captured during the annual trapping period (Jan-Mar 09). Trained staff members captured the males during the morning in a 100 m^2^ feeding corral provisioned with monkey chow, netting or capturing the monkeys by hand and transferring them subsequently to a field laboratory where they remained overnight. The following morning, animals were anaesthetized as described above.

All body measurements were collected within one hour of sedation. Body weight was determined to the nearest 0.1 kg using a platform scale (DPZ animals) or standard hanging scale (CS animals). For morphometric measurements, all animals were laid onto their left side in a standardized position with arms and legs perpendicular to the vertebral axis and the back fully straight [42]. We measured crown-rump length of each animal using a flexible tape measure with 1 mm gradations (DPZ animals) or 1 m ruler with 1 mm gradations (CS animals). For DPZ animals, we used a skinfold sliding calliper with 1 mm gradations to measure skinfold thickness (a measure of skinfold fat) in 5 body parts [43]: caudal aspect of upper arm (triceps), cranial aspect of thigh (quadriceps), belly lateral to the umbilicus, subscapular, and suprailiac. All skinfold measures were taken for the right side only and to the nearest millimetre. For CS animals, monkeys were placed on their side and measurements of skinfold thickness were taken from the belly approximately 2 cm above the umbilicus, using a Lange calliper accurate to 1 mm. For all measurements, we collected each measure three times and used a mean in analysis. Using morphometric data, we calculated the body mass index (BMI), by dividing body mass (kg) by crown-rump length squared (m^2^) [44]. In addition to the collection of morphometric data, a blood sample (2–4 ml) was collected from the femoral vein of each animal into a heparinised tube. Samples were stored on ice, centrifuged at 3000 rpm for 10 min and the plasma subsequently recovered and stored at −20°C until measurement. Samples from CS animals were shipped frozen to the endocrine laboratory of the Reproductive Biology Unit, DPZ, where all laboratory analyses were performed.

### Urine sample collection

For the majority of DPZ animals, urine samples could often not be collected on the day morphometric data were taken as animals usually emptied their bladder during capture. Instead, urine samples were collected from DPZ animals within three weeks following or preceding the collection of morphometric measures. Samples from DPZ animals living in outside enclosures were collected between 11:00 and 14:00, following a training period during which animals received a food reward in response to cognitive tests. For sample collection, plastic mats were placed below the tunnel used by the animals to travel from the inside to the outside compartment. Urine was collected from the mats directly following urination, placed on ice and stored within 3 hours at −20°C until C-peptide analysis. Samples from DPZ animals living in indoor cages were collected between 8:00 and 13:30 on plastic mats placed under the wire cage; these were stored within 6 hours at −20°C as described above.

During the 2–3 months before CS animals were trapped and measured, we collected 68 urine samples from the study males while they were free-ranging (see below for final sample sizes by male). Urine samples from CS animals were pipetted off the ground or other substrate (e.g. leaves, rocks) directly after a male was observed to urinate. Samples were collected between 7:20 and 13:40, but 78% of samples were collected between 7:20 and 10:20. Urine was placed into 2 ml Eppendorf safe-lock microcentrifugue tubes (VWR, West Chester, PA, USA) and placed on ice. The sample was checked for cleanliness, and if there was any particulate matter in the sample, this was allowed to settle to the bottom. The supernatant urine was then pipetted off into a fresh microcentrifuge tube. This process was repeated until the sample was clean. At the end of fieldwork for the day (either 11:30 or 14:30) samples were returned to the Carribean Primate Research Center (CPRC) field station on Puerto Rico, and frozen at −80°C until transportation on ice (together with the collected plasma samples) to the Reproductive Biology Unit, DPZ.

### Food reduction experiment

Using 6 of the indoor-housed captive adult macaques (the first 6 animals listed in Table 2), we conducted a feeding experiment, during which we controlled the amount of food provided. Prior to the onset of the experiment we determined the average amount of monkey chow being consumed daily by each individual over a period of 20 days. For this, individuals were isolated for feeding twice a day (09.15–10.15; 11.45–12.45) into single compartments of their home cage and we weighed the amount of monkey chow provided and the amount remaining after feeding. We then calculated the mean daily net intake over all 20 days (mean daily consumption). In addition, each individual was given one banana and one apple per day. For three days prior to the onset of the experiment we undertook a control period, in which each animal received their mean daily monkey chow consumption, plus the two fruits, in two feeding sessions. For the following two weeks, the amount of monkey chow given to each animal was restricted to 50% of the amount provided during the control period, and animals received in addition only half an apple and half a banana (diet period). Thereafter, animals were provisioned as during the control period (re-feeding period). Water was available *ad libitum* throughout the whole experiment. Before animals were reunited following feeding sessions, daily body weights were determined for each individual to the nearest of 0.05 kg using a platform scale. The scale was placed on the ground of the cage, and animals were trained to step on it and sit still until their weight measure was stable. During this experiment, urine samples were collected twice a day from each individual as described above. Morning samples were usually collected before animals received their first feed (fasting samples), while the second sample was usually collected 2–3 hours after the first feeding (non-fasting samples). Samples were placed on ice directly after collection and stored frozen at −20°C within 5 hours until analysis.

### C-peptide analysis

Prior to routine analysis, we tested on a few rhesus macaque urine samples the ability of two C-peptide ELISA Kits designed to measure C-peptide in human serum and plasma to detect C-peptide levels in macaque urine. One kit (DSL-10-7000) was purchased from DSL Diagnostic Systems Laboratories, Sinsheim, Germany, the other from IBL International GmbH, Hamburg, Germany (Art. No. RE 53011). While both assays were able to detect macaque urinary C-peptides levels well above assay sensitivity and levels measured in the two assays were significantly correlated (r = 0.72, p<0.05), concentrations measured with the IBL assay were 2-3 times higher than those measured in the DSL assay. Furthermore, urine sample dilutions ran parallel to the C-peptide standard curve in the IBL assay, while this was not clearly the case in the DSL assay. Thus, given the apparent higher cross-reactivity of the antibody used in the IBL assay with macaque C-peptide and the absence of interfering matrix effects in this assay compared to the one from DSL, we routinely analyzed all our urine samples using the IBL assay kit. Prior to assay, urine samples were diluted between 1∶2 and 1∶12 (depending on the C-peptide level and amount of urine available) with IBL sample diluent (Art. No. RE 53017) to bring the samples into the working range of the assay, and 100 µl of the diluted urine was then assayed using the manufacturer provided protocol. Assay sensitivity was 0.064 ng/ml. Inter-assay coefficients of variation calculated from the measurement of low, middle and high value quality controls run in each assay were 14.5%, 10.5% and 10.6%, respectively while intra-assay coefficients of variation values were 6.5%, 6.7% and 5.1%, respectively.

To adjust for differences in urine concentration, C-peptide values were indexed to the level of urinary creatinine measured according to the method described by Bahr and colleagues [45] and C-peptide concentrations are presented as ng C-peptide/mg creatinine. Means ± SE creatinine concentration (in mg/ml) per individual were: 0.39±0.04 for CS animals, 0.60±0.08 for captive animals outside of the experiment and 0.60±0.03 for DPZ animals used for the experiment (range for all individuals: min: 0.1, max: 3.27). The mean creatinine concentration was not affected by changes in animal diet and did not differ significantly across the different experimental phases (i.e. control, first week of diet, second week of diet, first week of re-feeding and second week of re-feeding) for the fasting samples (Friedman test, χ^2^ = 5.07, df = 4, p = 0.28) as well as for the non-fasting samples (χ^2^ = 5.07, df = 4, p = 0.81). Thus, adjusted C-peptide levels during the feeding experiment were not biased as a result of potential changes in creatinine concentrations.

Of the 328 samples collected for the DPZ animals and the 68 samples collected for CS animals, 6 and 32 samples, respectively, had values that were below C-peptide assay sensitivity. In these cases and as done by Deschner and colleagues [37], we assigned them the maximum possible value they could have taken, i.e. the value of assay sensitivity (0.064 ng/ml) so as not to artificially exclude samples with low C-peptide levels from analysis. Note that this is a very conservative approach to our data as it means that in samples of low (undetectable) concentration we have slightly overestimated their concentrations, so reducing variation in our dataset. We excluded one sample (from the CS animals) because of a low (<0.1 mg/ml) creatinine concentration (e.g. [46]). After samples from the same male but from different times on the same day were averaged, we were left with a dataset for CS animals of 64 different ‘male days’ (unique male day combinations), with a mean ± SE per male of 4.3±0.4 (range 2–12). For DPZ animals, the data set comprised 63 samples from 11 animals with a mean ± SE per individual of 5.7±1.5 (range 2–18). In addition 265 samples (185 fasting, 80 non-fasting) resulted from the feeding experiment on DPZ animals.

### Statistics

We examined the relationship between each individual's mean level of UCPs and: 1) BMI; 2) skinfold thickness; and 3) plasma C-peptide levels, using Spearman's correlations. Only animals for which at least two urine samples were collected were used for analysis. Data on captive and free-ranging animals were analyzed separately. As UCPs data for CS animals were skewed by two high outliers, we log-transformed all UCPs data for graphing. Note that as we used rank-based statistics, logging has no effect on results of our statistical tests. We used one-tailed probabilities, since we had clear predictions for a positive relationship between UCPs and each of the three variables tested and that the opposite effect would not have been expected.

For the feeding experiment, we examined the relationship between UCPs and body mass during the 28 days of the experiment using Spearman's correlations. Because levels of UCPs in fasting and non-fasting samples were strongly correlated (Spearmańs correlation: r_s_ = 0.88; p<0.001), we restricted the analysis of the effect of food availability and body mass changes on UCPs to fasting samples. For this, mean UCPs from the fasting samples and mean body mass were calculated across all individuals for a given day. In addition, to test the effect of food availability on UCPs, we divided the experimental period into 5 phases: control, diet 1 (first week of food reduction), diet 2 (second week of food reduction), re-feeding 1 (first week of re-feeding) and re-feeding 2 (second week of re-feeding). We tested for general differences in mean UCPs between the 5 experimental phases using the Friedman test. The Wilcoxon signed-ranks test was used post hoc to determine which of the phases differed significantly from each other. Because a study of bonobos [37] suggested that UCPs decrease during food reduction (diet period) and increase during food increase (re-feeding period), and because the opposite effect would not have been expected we used one tailed probabilities. Given the likely difficulties of collecting first morning for wild primates living in large groups (such as macaques) we tested whether UCPs relate to feeding condition (i.e. whether animals have or have not recently fed) independently of the time of sample collection. Accordingly, we used a Wilcoxon signed rank test to explore for difference between the mean individual UCP levels in fasting samples of the control, re-feeding 1 and re-feeding 2 periods (normal amount of food) and the mean individual UCP levels in non-fasting samples of the diet 1 and diet 2 periods (reduced food). Finally, we used a two-tailed Wilcoxon signed ranks test to explore differences in C-peptide levels between fasting and non-fasting samples.

All statistical tests were conducted with SPSS 15.0 for Windows or R 2.7.0, and we considered p<0.05 significant. For non parametric tests, exact p-values were computed whenever possible (where no ties occurred). Non-exact p-values are marked as “*” in the result section.

## Results

### UCPs, BMI, skin fatness and plasma C-peptide levels

**Figure 1:**
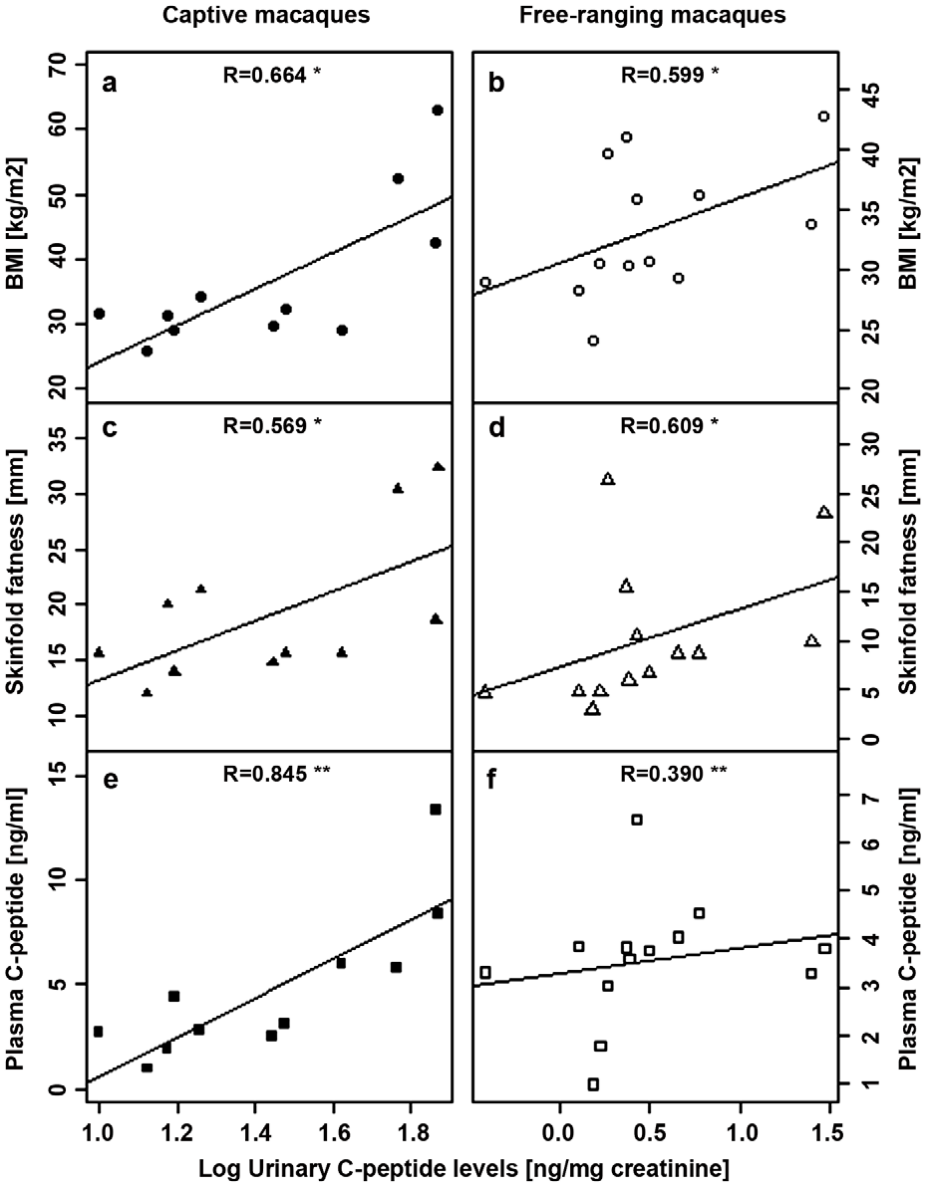
Individual UCP levels of animals in relation to BMI, skinfold fatness and plasma C-peptide levels. Mean individual log10 transformed UCPs of captive DPZ (left boxes; open symbols) and free-ranging Cayo Santiago (right boxes; black symbols) animals in relation to BMI (a,b), skinfold thickness (c,d) and plasma C-peptide levels (e,f). “*” P<0.05; “**”P<0.01. The UCP level values presented in this figure come from samples collected during periods of normal feeding; none come from the experimental period.

UCPs correlated positively with both BMI (DPZ animals: r_s_ = 0.664, p = 0.015, Figure 1a; CS animals: r_s_ = 0.599, p = 0.017*, Figure 1b) and skinfold thickness (DPZ animals: r_s_ = 0.569, p = 0.034, Figure 1c; CS animals: r_s_ = 0.609, p = 0.014*, Figure 1d). We found a strong and highly significant correlation between UCPs and plasma C-peptides for DPZ animals (r_s_ = 0.845, p<0.001, Figure 1e), but not CS animals (r_s_ = 0.390, p = 0.093, Figure 1f).

### UCPs in relation to body mass dynamics and dietary regime

**Figure 2:**
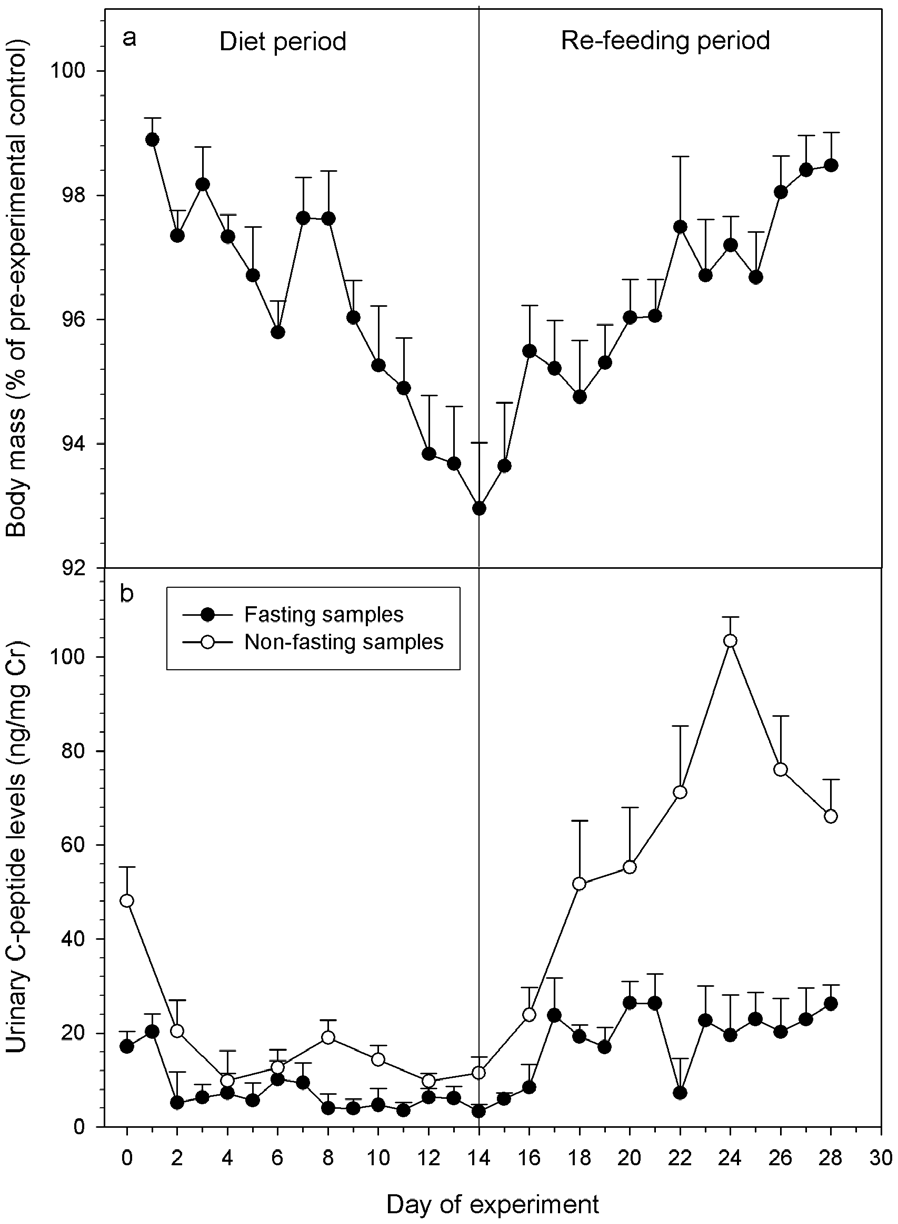
Changes in body mass and UCP levels during the feeding experiment. Changes in body mass (a) and UCP levels (b) for fasting (black circles) and non-fasting (white circles) samples during the diet and re-feeding period of the feeding experiment. For body mass, values represent percentages of the mean weight determined during the pre-experimental control period ( = 100%). All values represent medians with standard errors. C-peptide values on Day 0 represent individual mean levels during the pre-experimental control period.

**Figure 3:**
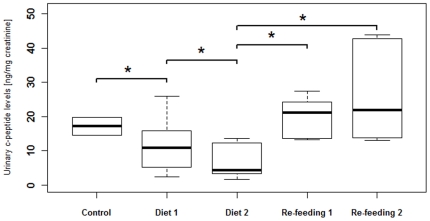
Concentration of UCP levels during the five phases of the feeding experiment. Box plots showing grouped concentrations of UCP levels, for the fasting samples, during the five phases of the food reduction experiment. The boxes indicate medians (line) and first and third quartiles. The whiskers indicate the 90th and 10th percentiles. “*” P<0.05

During the diet period of the food reduction experiment, animals lost an average 7.1% (range 4.3%–9.8%) of body mass (Fig. 2a). All animals gained weight during re-feeding (Fig. 2a). After the two week re-feeding period animals reached on average 98.2% (96.6%–100.1%) of their pre-experiment weight. As predicted, UCPs in fasting samples co-varied with body mass decreasing during the diet period, and increasing during the re-feeding period with non-fasting samples showing a bigger difference between diet and re-feeding period (in the latter even exceeding control period values) than fasting samples. Accordingly, we found a significant positive correlation between UCPs in fasting samples and body weight (r_s_ = 0.536, p<0.002). UCPs in fasting samples differed significantly across the 5 different experimental phases (χ^2^ = 19.6, df = 4, p<0.001; Figure 3), decreasing with food restriction (control vs. diet 1: Z = −2.201, p = 0.016; diet 1 vs. diet 2: Z = −2.201, p = 0.016; Figure 3) and increasing during re-feeding (diet 2 vs. re-feeding 1: Z = −2.201, p = 0.016; diet 2 vs. re-feeding 2: Z = -2.201, p = 0.016; Figure 3). Finally, UCPs in non-fasting samples were significantly higher than in fasting samples (Z = −6.619, p<0.001; Figure 2). However, UCPs in fasting samples of the control, re-feeding 1 and re-feeding 2 periods were still significantly higher than UCPs in non-fasting samples of the diet 1 and diet 2 periods (Z = −2.201, p = 0.031).

## Discussion

Our results demonstrate that urinary C-peptide is a useful biomarker of energy intake and body fatness not only in hominids [29], [33]–[37] but also in other primates (Table 1). UCP values were significantly correlated with BMI and body fatness (all animals), as well as plasma C-peptide levels (captive animals), showing that they on one hand reflect insulin production and on the other the animal's nutritional status. In addition to assessing these parameters during a given point in time, results of our feeding experiment show that measurement of UCPs also seems to be a valuable tool for tracking changes of individual diet and nutritional status over time.

A particular strength of our validation is the use of both captive and free-ranging macaques to demonstrate that relationships seen in captive animals can also be extrapolated into field settings, where animals usually have lower BMI and consequently also lower UCP levels. In general, results obtained in captive and free-ranging animals had the same predictive value in both populations. The failure of urinary and plasma C-peptide levels to correlate in the CS animals may be related to sample procedure. For CS animals, plasma C-peptide levels were collected from just one point in time, whereas mean UCP levels were developed from averages over a period of a few months prior to the collection of blood samples. Urine samples of captive animals, in contrast, were collected in a much narrower window around the day on which plasma samples were taken. More critical than this absence of correlation is that UCPs were below assay sensitivity in a portion of samples from several CS (and one DPZ) individuals. Since these problems occurred mainly in individuals at the lower end of the observed BMI variation and since low UCP levels seem less of a problem in studies of great apes which have larger bodies than macaques (e.g. [37]), body mass may principally be a critical parameter limiting the use of UCPs as biomarker. We were, however, able to measure very low C-peptide values in several individuals with low BMI in both free-ranging and captive animals as well as in juveniles (unpubl. data), suggesting that additional factors may be involved. Dilution of urine (in combination with low BMI) may play an important role here since in our study animals, water was available *ad libitum* and there may have been significant individual differences in water consumption. Applicability of UCP assays to field studies may therefore depend on availability of water and individual differences in drinking behaviour, as well as in the water content of food, and may thus vary between seasons and populations. In addition, environmental pollutants such as soil and faecal matter, may have contaminated urine samples and could potentially cause degradation or absorption of UCPs, particularly in samples taken from the ground (i.e. CS samples; see also [37]). Hence, further studies investigating the influence of sample contamination on UCP levels are important for optimizing sample collection procedures in the wild.

Complementary to our cross-sectional results, we also undertook a food-reduction experiment. The dietary restriction and re-feeding of macaques undertaken during our experiment revealed a close link between changes in C-peptide excretion, food intake and body mass, with UCP levels changing quickly (within two days) in response to changes in food supply and body weight. Given that C-peptide is a by-product of insulin production, changes in C-peptide excretion can be expected to indicate changes in insulin production. A tight relationship between food intake and UCPs has been found in field studies on great apes (i.e. [34], [36], see Table 1). Interestingly however, although food supply (and thus energy intake) remained constant during the food restriction period, UCP levels further decreased significantly as body mass continued to decline. This indicates that UCP excretion is not just sensitive to caloric intake (i.e. is not just a measurement of dietary input), but also to body mass itself.

In our study, non-fasting samples contained higher UCPs than fasting samples; food consumption via stimulating insulin production thus seems to have a direct effect on UCP levels which in turn may confound their reliability when assessing inter-individual differences in the energetic status of wild animals. The collection of urine at standardized times of the day (e.g. at early morning sleeping sites before animals have moved) should help to control for such effects. For species living in large groups, however, collection of samples at standardized times may be impossible. Nevertheless, we found that average UCP levels after feeding (i.e. non-fasting samples) under reduced food conditions were significantly lower than fasting UCPs under normal feeding, demonstrating the potential of UCPs to assess changes in energy intake regardless of sample collection time. Furthermore, animals feed more continuously in the wild than under most captive conditions, and short term variation in UCPs is likely to be less dramatic in wild animals than in captive ones with fixed feeding times and highly calorific food.

Our results are in line with previous studies on humans and great apes (Table 1) demonstrating that UCP levels show a positive relationship with measures of individual nutritional status (humans: [29], [33]; bonobos: [37]) and food intake (humans: [33]; bonobos:[37]; orang-utans: [36]). They are also in line with a previous study on rhesus macaques demonstrating that obese animals have higher levels of UCP excretion than non-obese macaques and that complete food deprivation leads to dramatic decrease in UCP levels [38] (see also Table 1). More importantly, they extend this finding considerably by showing that less extreme body mass differences and dietary changes are reflected in C-peptide excretion. In the present study, animals lost on average only 7% of body mass, which was associated with a clear reduction in UCP levels. Macaques regularly demonstrate this type of intra-individual body mass variation; for example, male bonnet macaques lose 6–8% and male rhesus macaques 10–12% of body weight during the mating season due to costly reproductive strategies [47]–[49]. We thus show that UCPs are sensitive enough to track the amount of body mass variation typically associated with the behavioural and reproductive strategies seen in macaques. Furthermore, we show that UCP measurements are viable markers of nutritional status from single void urine samples and that it is unnecessary to collect complete 12 hour samples (as done by Wolden-Hanson and colleagues, [38]). This is consistent with results recently presented for non-human great ape species [34]–[37] and makes UCP measurement more applicable to field studies.

In our study, loss of body mass was induced by a reduction of energy intake; energy balance is however usually a product of both energy intake and expenditure. To date, only one study has taken energy expenditure into account when examining UCP levels. In a study on colobus monkeys, Harris and colleagues [50] showed that UCPs are positively correlated with the distance travelled by females 24 h before sample collection. Although it is unclear how reliably UCPs reflect blood C-peptide levels and individual energetic condition in colobus monkeys, the results of our study suggest that the use of UCPs as a marker of energetic condition in this species is likely to be justified. Further studies measuring both energetic expenditure and intake as well as UCP levels from free-ranging primates will improve our understanding of the relationships between the three parameters.

In summary, our study provides the first validation of the use of UCP levels as a non-invasive measure of body fatness and nutritional status in a non-great ape primate species, and suggests that UCPs are a useful biomarker for monitoring changes in nutritional status in studies of primates (and possibly also other mammals) more generally. Potential uses of this biomarker span a broad range of topics such as studies of aging and reproduction, food competition, effects of stress and season, and behavioural strategies. We therefore encourage additional validation, evaluation and adjustment of assays for UCP measurement in other mammalian taxa. This tool opens up exciting new opportunities for field studies in ecology (e.g. on the influence of habitat structure/home range size/group size/availability of specific resources and so on on individual nutritional status), behavioural ecology (e.g. on the costs of food competition and on the relationship between dominance status and age on nutritional status) and reproductive biology (e.g. on the link between nutritional status and reproductive output, and on the costs of specific reproductive strategies). However, before measurement of UCPs is fully applied to field settings, certain issues related to the collection and storage of samples (e.g. effects of sample contamination by soil/faeces, transportation related freeze-thaw and so on), should be systematically investigated. Finally, given that c-peptides are generally produced in equimolar amounts to insulin, and given the positive relationship we found between serum c-peptide levels and UCP levels, UCP measurement may also be a useful non-invasive tool in clinical studies.

### Conclusion

Our study shows that C-peptide levels measured non-invasively from urine samples reflect changes in body mass, body fatness and food intake in captive and free-ranging macaques. Our results thus validate for the first time the utility of UCPs as reliable biomarkers of nutritional status in a non-hominid primate species, and suggest that the measurement of UCPs has potential for being useful for a broader range of mammals. This new biomarker opens up exciting opportunities for studies of ecology, behavioural ecology and reproductive biology, and also for biomedical studies under captive, free-ranging and natural settings.

## References

[1] Lindström J (1999). Early development and fitness in birds and mammals.. Trends in Ecology & Evolution.

[2] Bronson FH (1989). Mammalian Reproductive Biology..

[3] Cuthill IC, Houston AI, Krebs JR, Davies NB (1997). Managing time and energy.. Behavioural ecology: an evolutionary approach.

[4] Jürgens KD, Prothero J (1991). Lifetime energy budgets in mammals and birds.. CompBiochemPhysiol.

[5] Karasov WH (1986). Energetics, physiology and vertebrate ecology.. Trends in Ecology & Evolution.

[6] Krebs JR, Davies NB (1993). An Introduction to Behavioural Ecology..

[7] Berman CM, Schwartz S (1988). A non-intrusive method for determining relative body-fat in free-ranging monkeys.. Am J Primatol.

[8] Koenig A, Borries C, Chalise MK, Winkler P (1997). Ecology, nutrition and timing of reproductive events in an Asian primate, the Hanuman langur (*Presbytis entellus*).. J Zool.

[9] Altmann J, Schoeller D, Altmann SA, Muruthi P, Sapolsky RM (1993). Body size and fatness of free-living baboons reflect food availability and activity levels.. Am J Primatol.

[10] Goldizen AW, Terborgh J, Cornejo F, Porras DT, Evans R (1988). Seasonal Food Shortage, Weight Loss, and the Timing of Births in Saddle-Back Tamarins (Saguinus fuscicollis).. J Anim Ecol.

[11] Puri CP, Puri V, Anand Kumar TC (1981). Serum levels of testosterone, cortisol, prolactin and bioactive luteinizing hormone in adult male rhesus monkeys following cage-restraint or anaesthetizing with ketamine hydrochloride.. Acta Endocrinol.

[12] Sapolsky RM (1982). The endocrine stress-response and social status in the wild baboon.. Horm Behav.

[13] Sapolsky RM (1985). Stress-induced suppression of testicular function in the wild baboon: role of glucocorticoids.. Endocrinology.

[14] Sapolsky RM (1986). Stress induced elevation of testosterone concentrations in high ranking baboons: role of catecholamines.. Endocrinology.

[15] Altmann J, Alberts SC (1987). Body mass and growth rate in a wild primate population.. Oecologia.

[16] Mori A (1979). Analysis of population changes by measurement of body weight in the Koshima troop of Japanese monkeys.. Primates.

[17] Altmann S (1998). Foraging for Survival..

[18] Chivers DJ (1998). Measuring food intake in wild animals: primates.. Proc Nutr Soc.

[19] Altmann J, Samuels A (1992). Costs of maternal care: infant-carrying in baboons.. Behav Ecol Sociobiol.

[20] Barton RA, Whiten A, Byrne RW, English M (1993). Chemical composition of baboon plant foods: implications for the interpretation of intra- and interspecific differences in diet.. Folia Primatol.

[21] Schülke O, Chalise MK, Koenig A (2006). The importance of ingestion rates for estimating food quality and energy intake.. Am J Primatol.

[22] Knott CD (1998). Changes in orangutan caloric intake, energy balance, and ketones in response to fluctuating fruit availability.. Int J Primatol.

[23] Kelly TR, Sleeman JM, Wrangham RW (2004). Urinalysis in free-living chimpanzees (Pan troglodytes schweinfurthii) in Uganda.. The Veterinary Record.

[24] Knott CD, Brockman D, van Schaik CP (2005). Energetic responses to food availability in the great apes: Implications for hominin evolution.. Primate seasonality: Implications for human evolution.

[25] Wich SA, Utami-Atmoko SS, Mitra Setia T, Djoyosudharmo S, Geurts ML (2006). Dietary and energetic responses of *Pongo abelii* to fruit availability fluctuations.. Int J Primatol.

[26] Blanc S, Colman R, Kenmitz J, Weindruch R, Baum S (2005). Assessment of nutritional status in rhesus monkeys: comparison of dual-energy X-ray absorptiometry and stable isotope dilution.. J Med Primatol.

[27] Lifson N, McClinto R (1966). Theory of use of turnover rates of body water for measuring energy and material balance.. J Theor Biol.

[28] Rubenstein D, Ymashita S, Melmed S (1969). Secretion of proinsulin C-peptide by pancreatic bold beta cells and its circulation in blood.. Nature.

[29] Kruszynska YT, Home PD, Hanning I, Alberti K (1987). Basal and 24-h C-peptide and insulin secretion rate in normal man.. Diabetologia.

[30] Meistas MT, Foster GV, Margolis S, Kowarski AA (1982). Integrated concentrations of growth hormone, insulin, C-peptide and prolactin in human obesity.. Metabolism.

[31] Hoffman CL, Ruiz-Lambides AV, Davila E, Maldonado E, Gerald MS (2008). Sex differences in survival costs of reproduction in a promiscuous primate.. Behav Ecol Sociobiol.

[32] Yoshida NM, Yoshiuchi K, Kumano H, Sasaki T, Kuboki T (2006). Changes in heart rate with refeeding in anorexia nervosa: a pilot study.. J Psychosom Res.

[33] Hoogwerf BJ, Laine DC, Greene E (1986). Urine C-peptide and creatinine (Jaffe method) excretion in healthy young adults on varied diets: sustained effects of varied carbohydrate, protein, and meat content.. Am J Clin Nutr.

[34] Emery Thompson M, Muller MN, Wrangham RW, Lwanga JS, Poots KB (2009). Urinary C-peptide tracks seasonal and individual variation in energy balance in wild chimpanzees.. Horm Behav.

[35] Sherry DS, Ellison PT (2007). Potential applications of urinary C-peptide of insulin for comparative energetics research.. Am J Phys Anthropol.

[36] Emery Thompson M, Knott CD (2008). Urinary C-peptide of insulin as a non-invasive marker of energy balance in wild orangutans.. Horm Behav.

[37] Deschner T, Kratzsch J, Hohmann G (2008). Urinary C-peptide as a method for monitoring body mass changes in captive bonobos (*Pan paniscus*).. Horm Behav.

[38] Wolden-Hanson T, Davis GA, Baum ST, Kenmitz JW (1993). Insuline levels, physical activity and urinary catecholamine excretion of obese and non obese rhesus monkeys.. Obes Res.

[39] Harris TR, Chapman CA, Monfort SL (2010). Small folivorous primate groups exhibit behavioral and physiological effects of food scarcity.. Behav Ecol.

[40] Rawlins RG, Kessler MJ (1986). The Cayo Santiago Macaques..

[41] Marriott BM, Roemer J, Sultana C (1989). An overview of the food intake patterns of the Cayo Santiago rhesus monkeys (Macaca mulatta): report of a pilot study.. Puerto Rico Health Sciences Journal.

[42] Muehlenbein MP, Campbell BC, Richards RJ, Watts DP, Svec F (2005). Leptin, adiposity, and testosterone in captive male macaques.. Am J Phys Anthropol.

[43] Hamada Y, Hayakawa S, Suzuki J, Ohkura S (1999). Adolescent growth and development in Japanese macaques (Macaca fuscata): Punctuated adolescent growth spurt by season.. Primates.

[44] Campbell BC, Gerald MS (2004). Body composition, age and fertility among ree-ranging female rhesus macaques (*Macaca mulatta*).. J Med Primatol.

[45] Bahr NI, Palme R, Möhle U, Hodges JK, Heistermann M (2000). Comparative aspects of the metabolism and excretion of cortisol in three individual nonhuman primates.. Gen Comp Endocr.

[46] Muller MN, Wrangham RW (2004). Dominance, aggression and testosterone in wild chimpanzees: a test of the ’challenge hypothesis‚.. Anim Behav.

[47] Bernstein IS, Weed JL, Judge PG, Ruehlmann TE (1989). Seasonal weight changes in male rhesus monkeys (Macaca mulatta).. Am J Primatol.

[48] Cooper MA, Chaitra MS, Singh M (2004). Effect of dominance, reproductive state, and group size on body mass in *Macaca radiata*.. Int J Primatol.

[49] Glick BB (1979). Testicular size, testosterone level, and body-weight in male *Macaca-radiata*- maturational and seasonal effect.. Folia Primatol.

[50] Harris TR, Montfort SL (2007). Evaluating c peptide as a measure of net energy gain in Colobus guereza.. Am J Phys Anthropol.

